# Modulation of Peripheral Immune Cells Following Vitamin D_3_ Supplementation in Vitamin D-Insufficient Cancer Patients

**DOI:** 10.3390/nu18010116

**Published:** 2025-12-29

**Authors:** Alexandra Kalmar, Zsofia Brigitta Nagy, Liza Dalma Sumegi, Barbara Kinga Bartak, Csaba Kiss, Sandor Spisak, Bela Molnar, Istvan Takacs

**Affiliations:** 1 Department of Internal Medicine and Oncology, Semmelweis University, 1083 Budapest, Hungary; kalmar.sopkez.alexandra@semmelweis.hu (A.K.); nagy.zsofia@semmelweis.hu (Z.B.N.); sumegi.liza.dalma@semmelweis.hu (L.D.S.); molnar.barbara.kinga@semmelweis.hu (B.K.B.); molnar.bela@semmelweis.hu (B.M.); 2 Institute of Molecular Life Sciences, HUN-REN Research Centre for Natural Sciences, 1117 Budapest, Hungary; kiss.csaba1173@gmail.com (C.K.); spisak.sandor@ttk.hu (S.S.); 3 Doctoral School of Biology, Eötvös Loránd University, 1117 Budapest, Hungary

**Keywords:** vitamin D, cancer, ATAC-Seq, chromatin accessibility, Comet-assay, PBMC

## Abstract

**Background and aims**: Low vitamin D_3_ levels are common in cancer patients, and these patients might benefit from vitamin D_3_ level normalization in parallel with the conventional oncology treatment. This study aimed to examine the molecular effects of moderate–high-dose vitamin D_3_ supplementation in vitamin D-deficient cancer patients. **Methods**: Eight patients under oncological treatment (5 lung cancer, 2 colorectal cancer, and 1 urothelial carcinoma) received 30,000 IU of vitamin D_3_ per week for two months. Blood samples were collected before and after supplementation, and peripheral blood mononuclear cells (PBMCs) were isolated. With the aim of assessing further potential epigenetic alterations, global DNA methylation level was estimated on the basis of LINE-1 bisulfite-sequencing experiments on cfDNA and PBMC cells. In order to explore the chromatin accessibility alterations after the treatment in PBMCs, an assay for transposase-accessible chromatin with sequencing (ATAC-Seq) was performed using the (10x Genomics, Pleasanton, CA, USA) on a NextSeq 550 instrument using High Output Sequencing kit (Illumina, San Diego, CA, USA). DNA integrity was assessed by the alkaline Comet-assay and telomere qPCR was also performed. **Results**: After serum 25-hydroxy-vitamin D levels were normalized, DNA integrity in mononuclear cells improved significantly (*p* = 0.01), while no significant changes were found in granulocytes. Vitamin D_3_ supplementation also led to significant changes in telomere length in mononuclear cells (*p* = 0.007). No significant differences were observed in cfDNA levels or DNA methylation in PBMCs and cfDNA after supplementation. ATAC-Seq revealed changes in PBMC composition, including an increased number of NK, pDC cells, and monocytes, especially in patients treated with Pembrolizumab in parallel with vitamin D supplementation. **Conclusions**: These exploratory findings suggest that the observed immune cell and chromatin changes after vitamin D_3_ level normalization are compatible with immunomodulatory effects and warrant confirmation in larger, controlled cohorts.

## 1. Introduction

Vitamin D is essential for normal immune function [1]. Upon exposure to UV-B radiation, vitamin D_3_ is produced in a non-enzymatic reaction from 7-dehydrocholesterol in the skin [2]. Vitamin D must be converted into its active hormonal forms, 25-hydroxyvitamin D (25OHD) and 1,25-dihydroxyvitamin D (1,25(OH)_2_D), by CYP enzymes in the liver and kidneys, respectively [3]. Innate immune system cells, including monocytes, macrophages, and dendritic cells, express CYP27B1, which enables these cells to produce 1,25(OH)_2_D_3_ for autocrine and paracrine functions [4]. The vitamin D receptor (VDR) is expressed across all immune cell types, highlighting vitamin D’s regulatory role in immunity [5]. Vitamin D influences immune cell differentiation, metabolism, maturation, and cytokine responses [6,7]. It enhances innate immunity while modulating adaptive responses by promoting anti-inflammatory and tolerogenic environments, thereby limiting excessive inflammation. These effects suggest a role for vitamin D in combating infections and influencing tumor development and progression [8,9,10]. However, the exact molecular mechanisms by which 1,25-dihydroxyvitamin D_3_ exerts immunomodulatory effects during a 2 months supplementation period remain to be fully explored. Studies have shown that vitamin D metabolites contribute to the regulation of proliferation, apoptosis, autophagy, antioxidant defense, and DNA repair during cancer formation and have chemoprotective effects [11].

Several observational and randomized clinical trials have found an association between serum 25-hydroxyvitamin D (25-OH-D) levels and cancer mortality. Observational studies have established that lower serum vitamin D levels are associated with higher overall cancer mortality [12,13,14,15,16]. A meta-analysis of 12 cohort studies found a 14% higher cancer mortality among individuals with the lowest 25-OH-D levels compared with those with the highest levels [14]. Similarly, the Prostate, Lung, Colorectal, and Ovarian Cancer Screening Trial reported a 17% lower cancer mortality among men and women in the highest vitamin D category compared with the lowest category [17]. Another meta-analysis of randomized controlled trials using higher doses of vitamin D (>1100 IU/day) found a 13% reduction in cancer mortality over three to ten years of follow-up [18]. Recent large observational studies support an association between higher serum 25-OH-D levels and reduced cancer-specific and all-cause mortality [19], with deficiency linked to increased mortality in population-based cohorts [20]. A meta-analysis further confirmed inverse associations between 25-OH-D and cancer mortality [21]. Evidence from randomized trials is mixed; however, higher-dose vitamin D (>1100 IU/day) supplementation was associated with a 13% reduction in cancer mortality [18], and updated individual patient data meta-analyses suggest daily vitamin D_3_ may modestly reduce cancer mortality depending on dosing regimen [22]. Although supplementation may have population-wide potential—for example, Niedermaier et al. estimated that vitamin D supplementation in older adults could prevent up to 30 000 cancer deaths annually in Germany [23]—randomized controlled trials to date have not demonstrated a preventive effect of vitamin D supplementation on cancer incidence despite suggestive findings for reduced cancer mortality in secondary analyses [24].

The molecular background of the long-term immune mechanisms underlying the reduced risk of tumor mortality is only partially understood. In humans, 1,25(OH)_2_D acts as a nuclear hormone that can activate the vitamin D receptor (VDR) transcription factor at sub-nanomolar concentrations [25,26]. Thus, it acts as a transcription factor that controls the expression of several vitamin D target genes [27]. The primary effect of vitamin D depends on VDR activation, which leads to epigenetic changes that modify the chromatin landscape and influence the transcriptional activity of target genes. Therefore, 1,25(OH)_2_D acts as an endocrine regulator of diverse cellular processes, including chromatin remodeling, and enhances intestinal absorption of the essential dietary mineral phosphorus [28]. Emerging research has associated dysregulated phosphorus homeostasis with tumorigenesis, raising the possibility that reduced vitamin D levels may contribute to altered phosphorus absorption in cancer [29].

Techniques such as FAIRE-Seq or ATAC-Seq can be used to determine accessible chromatin loci of vitamin D target genes [30]. However, chromatin accessibility data from real clinical oncology patients with vitamin D deficiency after vitamin D_3_ supplementation are still lacking. More than 1000 genes are known primary targets of VDR and 1,25(OH)_2_D; their expression alters within a few hours of VDR activation [31]. However, the impact of the normalization of vitamin D deficiency causes in immune cells long term, which could be of particular importance in terms of anti-tumor effects remains to be explored.

Peripheral blood mononuclear cells (PBMCs) are a mixture of cells, including monocytes, T cells, and B cells. Monocytes are likely the most vitamin D-responsive component, responding to vitamin D stimulation at more than 500 promoter and 2500 enhancer loci [25].

These observations highlighted the immuno-oncological aspects of vitamin D, prompting us to investigate genome-wide, vitamin D–dependent chromatin accessibility changes. We evaluated genomic and epigenetic effects by performing comet assays and telomere length analyses in monocytes and granulocytes, and assessing global DNA methylation in these cells as well as in plasma-derived cfDNA. This is the first clinical study to perform single-cell ATAC-Seq on peripheral blood mononuclear cells from severely vitamin D–deficient oncology patients before and after supplementation. The primary aim was to assess immune cell alterations potentially influencing cancer response. We hypothesize that, in this exploratory pilot study, restoring sufficient vitamin D levels in vitamin D–insufficient patients may lead to favorable molecular and immunological outcomes, including telomere elongation, reduced DNA damage, decreased cfDNA, improved global methylation, and altered chromatin accessibility responses.

## 2. Materials and Methods

### 2.1. Patients

Eight male patients with vitamin D deficiency and histologically confirmed solid tumors were included in this study. The median age was 63 years (range: 48–74), and all participants had an Eastern Cooperative Oncology Group (ECOG) performance status of 0 or 1 at baseline, indicating maintained functional ability. Tumor types included pulmonary adenocarcinoma (*n* = 4), small cell lung cancer (SCLC; *n* = 1), colorectal adenocarcinoma (*n* = 2), and high-grade muscle-invasive urothelial carcinoma with synchronous prostate adenocarcinoma (*n* = 1). All patients received standard oncologic treatments according to the current clinical guidelines. These included platinum-based chemotherapy regimens (e.g., cisplatin-etoposide, carboplatin-paclitaxel, gemcitabine-cisplatin), immune checkpoint inhibitors (pembrolizumab), and surgical resection when indicated. Radiotherapy was administered in selected cases, such as mediastinal and pelvic irradiation. Patients receiving immune checkpoint inhibitor therapy were analyzed separately, taking into account the expected immunomodulatory effect of the treatment. As a limitation of this pilot study, a non-supplemented control group could not be included because, based on our prior experience, as vitamin D supplementation is safe and has only beneficial effects in vitamin D–deficient patients [32]. Molecular profiling was available for all patients with pulmonary adenocarcinoma. *KRAS* mutations were detected in two cases, including one with a *KRAS* p.G12C variant. *EGFR*, *ALK*, *ROS1*, and *BRAF* mutations were negative in all tested patients. PD-L1 expression ranged from 1% to 60%, with three patients showing ≥40% expression. One patient had a concurrent benign Warthin tumor, and another presented with intracranial metastases.

All patients voluntarily provided blood samples before and after receiving oral vitamin D_3_ supplementation at a dose of 30,000 IU per week for a duration of two months, a regimen that has been shown to have a favorable safety profile in previous clinical studies [32,33]. During the study period, participants were monitored for safety, and no clinically significant adverse events or signs of vitamin D intoxication were detected. No changes were made to their oncologic therapy during the supplementation period. The study was conducted between March and May 2022, a time of year when vitamin D deficiency is particularly prevalent in Hungary [34,35]. Blood collection and laboratory analyses were performed at the Department of Internal Medicine and Oncology, Semmelweis University, Budapest, Hungary. Vitamin D deficiency was defined as serum 25(OH)D levels below 20 ng/mL. The study was approved by the National Medical Research Council (ETT TUKEB 22909-6/2021; approved date: 7 May 2021). Detailed patient information is provided in Table 1.

### 2.2. Isolation of PBMC Cells and Nuclei

PBMCs were isolated with the Histopaque method. First round centrifugation was performed at 1350 rcf for 12 min, then plasma samples were transferred to tubes and centrifuged again. Plasma samples were stored at −20 °C for later use. The cell fraction was adjusted with Dulbecco Saline Buffer to 10 mL (Sigma-Aldrich (St. Louis, MO, USA)) and was carefully transferred onto Histopaque layers (3 mL Histopack 1119 at the bottom, 3 mL 1077 to the top) (Sigma Aldrich). Centrifugation was performed at 700 rcf for 30 min and mononuclear and granulocyte cell fractions were collected to new tubes. After double washes with DPBS, centrifuged at 200 rcf for 10 min, live cell count was measured by using Countess II FL Automated Cell Counter according to the manufacturer’s instructions (Thermo Fisher Scientific, Waltham, MA, USA). Red blood cell lysis was performed by using 1× Miltenyi RBC Lysis Buffer (Miltenyi Biotec, Bergisch Gladbach, Germany) for 5 min at RT. Cells were sedimented at 300 rcf for 10 min, then a washing step was performed with DPBS followed by 300 rcf for 10 min. DNAse I RNase-free (1 U/μL, Thermo Fisher Scientific) treatment was performed with 300 μL DNase solution according to the protocol of Nuclei Isolation for Single Cell ATAC Sequencing (10x Genomics, Pleasanton, CA, USA). Flowmi cell strainer (Bel-Art Products, Wayne, NJ, USA) (40 μm) was applied to filter the cell suspension. Nuclei isolation was performed with 106 cell number input with 0.1× Lysis Buffer applied for 2 min according to Chromium Next GEM Single Cell ATAC Reagent Kits v1.1 protocol (10x Genomics). Nuclei were counted by Countess II and nuclear membrane integrity was assessed by microscope (40× magnification).

### 2.3. Comet Assay

Comet assays were performed by using Abcam Comet kit (Abcam, Cambridge, UK) according to the manufacturer’s instructions. Briefly, PBMCs and granulocytes were added to melting point agarose in 10% ratio, then this mix was pipetted on glass slides coated with melting point agarose (1%). After 15 min chill on 4 °C, slides were incubated in lysis buffer for 40 min, then in alkaline buffer for 30 min. Electrophoresis was performed in pre-chilled electrophoresis solution with 1 V/cm (300 mA, 25 V) for 45 min (nanoPAC-300P, Cleaver Scientific, Rugby, UK). This was followed by 3 × 2 min rinse in distilled water at 4 °C, then cells were fixed in 70% EtOH for 5 min. Drying was completed O/N, and SybrGOLD DNA dye (1:10,000 diluted in TE) was applied for 15 min. Mounting was performed with Dako Fluorescence medium (Dako, Glostrup, Denmark). Approximately 200 cells were documented with an inverse microscope using fluorescent filters at 40× magnification. Cometscore 2.0 software was used to detect the following parameters: tail DNA%, head DNA%, tail moment, and olive tail moment.

### 2.4. Telomere qPCR

The protocol of the Absolute Human Telomere Length Quantification qPCR Assay Kit (AHTLQ) (Catalog #8918, ScienCell Research Laboratories, Carlsbad, CA, USA) was followed according to the manufacturer’s instructions. Briefly, for each standard and genomic DNA sample, two 20 μL qPCR reactions were prepared, one with the telomere primer stock and one with the SCR primer stock, in triplicate. PCR plates were sealed and centrifuged at 1500× *g* for 15 s. Follow the qPCR program setup with an annealing temperature of 52 °C, with 10 min of initial denaturation at 95 °C, followed by 32 cycles of 20 s denaturation at 95 °C, 20 s annealing at 52 °C, and 45 s extension at 72 °C. Data acquisition occurred after each extension step.

### 2.5. Circulating DNA Isolation

Cell-free nucleic acids were extracted from the plasma samples of all patients using the Quick-cfDNA Serum & Plasma Kit (Zymo Research, Irvine, CA, USA). The quantity of the extracted nucleic acids was measured using the Qubit HS dsDNA Kit (Invitrogen, Waltham, MA, USA), and quality assessment was performed using the BioAnalyzer 2100 microcapillary electrophoresis system with High Sensitivity DNA Chips (Agilent Technologies, Santa Clara, CA, USA).

### 2.6. Global DNA Methylation Analyses

LINE-1 bisulfite sequencing was performed as described before. Briefly, 500 ng of DNA was isolated from cells, and approximately 20 ng of cfDNA was bisulfite converted by using the EZ DNA Methylation-Direct Kit (Zymo Research) according to the manufacturer’s protocol. Bisulfite-specific PCR was performed with Pyromark Q24 CpG LINE-1 Kit (Qiagen, Hilden, Germany) using the following thermocycling program: 15 min 90 °C; 45 cycles of 30 s 94 °C, 30 s on 50 °C and 30 s on 72 °C; 10 min final extension on 72 °C. After agarose gel (2%) specificity check, amplicons were prepared on the PyroMark Q24 Vacuum Workstation (Qiagen) and sequenced on Pyromark Q24 (Qiagen) instrument using Pyromark Q24 CpG LINE-1 Kit (Qiagen) and PyroMark Gold Q24 Reagents (Qiagen). Altogether 3 CpG sites (positions 318, 321, and 328 of LINE-1, GenBank accession number: X58075) were analyzed and the average methylation level was calculated.

### 2.7. Single-Cell ATAC-Seq

Single-cell libraries were constructed by using the Chromium Next GEM Single Cell ATAC Reagent Kits v1.1 protocol (10x Genomics) according to the manufacturer’s instructions. Briefly, transposition and GEM generation & barcoding were performed by using the droplet microfluidics-based Chromium Controller (10x Genomics). This step was followed by PostGEM incubation cleanup steps using Dynabeads and SPRIselect. Library construction was performed with individual Single Index N Set A to each sample by applying the following thermal cycling conditions: 98 °C for 45 s, 9 cycles (with the targeted recovery of 7000 nuclei) of 98 °C for 20 s, 67 °C for 30 s and 72 °C for 20 s, then 4 °C hold. Double-sided size selection was performed by using SPRIselect, followed by post-library construction QC using the KAPA Library Quantification Kit for Illumina Platforms (Roche Diagnostics, Basel, Switzerland) and the BioAnalyzer High Sensitivity Chip (Agilent) on a Bioanalyzer 2100 instrument.

### 2.8. Sequencing

Chromium Single Cell ATAC Libraries were paired-end sequenced by using NextSeq 500/550 High Output sequencing kit (Illumina, San Diego, CA, USA) according to the manufacturer’s protocol with 1.7 pM loading concentration and 1% PhiX.

### 2.9. Bioinformatic Analysis

Downstream analysis was conducted in R (v4.2.2) using the ArchR framework (v1.0.2). Quality control was applied by excluding cells with a transcription start site (TSS) enrichment score < 4 and unique fragment counts < 1000 to remove low-quality nuclei and debris. Doublets were removed using ArchR’s doublet scoring system. Dimensionality reduction and batch correction were performed using iterative Latent Semantic Indexing (LSI) with Harmony integration to correct patient-specific batch effects. Single-cell clustering was based on reduced-dimensional embeddings, and clusters were visualized using uniform manifold approximation and projection (UMAP). Cluster annotation was guided by hematopoietic scRNA-seq reference data from Granja et al. (2019) [36].

For differential accessibility testing between pre- and post-supplementation groups, the Wilcoxon rank-sum test was employed. This non-parametric test was selected due to the sparsity and non-normal distribution inherent in single-cell chromatin accessibility data. To ensure robust statistical power, a minimum threshold of 50 cells per sample was applied for inclusion in differential analyses. This minimum ensures sufficient aggregate signal to distinguish true biological variability from technical noise and dropout events common in scATAC-seq data [37]. Consequently, Patient #3 was omitted from the comparative analysis due to insufficient cell recovery following supplementation. Correction for multiple hypothesis testing was performed using the Benjamini–Hochberg procedure; features with a False Discovery Rate (FDR) ≤ 0.05 and |Log2FC| ≥ 1 were considered statistically significant.

## 3. Results

### 3.1. Vitamin D Level After 2 Months of Supplementation in Oncology Patients

**Figure 1:**
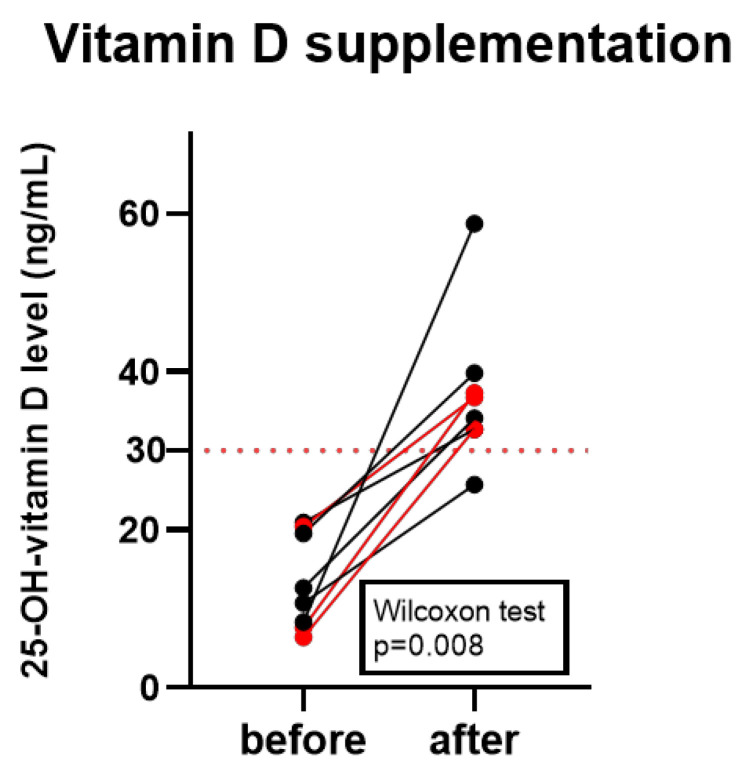
Vitamin D levels before and after treatment of oncology patients. Serum 25(OH)D levels elevated significantly (Wilcoxon test, *p* = 0.008) after the supplementation period. Red datapoints highlight pembrolizumab-treated patients.

Serum 25(OH)D levels significantly increased following treatment with 30,000 IU of vitamin D_3_ supplementation per week for 2 months (before: 13.3 ± 6.08 ng/mL vs. after: 37.21 ± 9.65 ng/mL) (*p* = 0.008). All participants reached sufficient serum 25(OH)D concentrations (≥30 ng/mL) during the supplementation period, except for one colorectal cancer patient, whose vitamin D level also improved considerably (Figure 1).

### 3.2. Comet Assay

**Figure 2:**
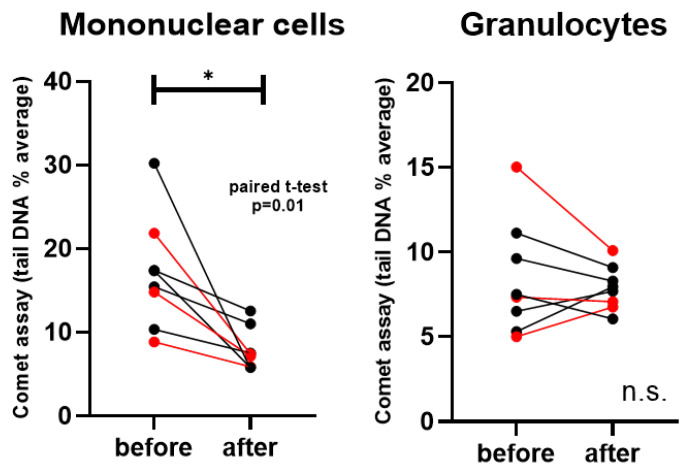
Comet assay (tail length % averages) in mononuclear cells and granulocytes. Red datapoints highlight pembrolizumab-treated patients. * *p* = 0.01; n.s. = not significant.

According to the Comet assay, DNA-tail average % was significantly lower after the treatment in the mononuclear cells (Paired *t*-test, *p* = 0.01), meaning higher overall DNA integrity in vitamin D sufficient patients. In all patients, regardless of the origin of tumors, mononuclear cells were found to have lower comet tails after the supplementation period. Interestingly, one of the CRC patients showed the highest decrease in mononuclear cell comet tail DNA% average. No significant alteration was detected in granulocytes. (Figure 2).

### 3.3. Telomere Length qPCR

**Figure 3:**
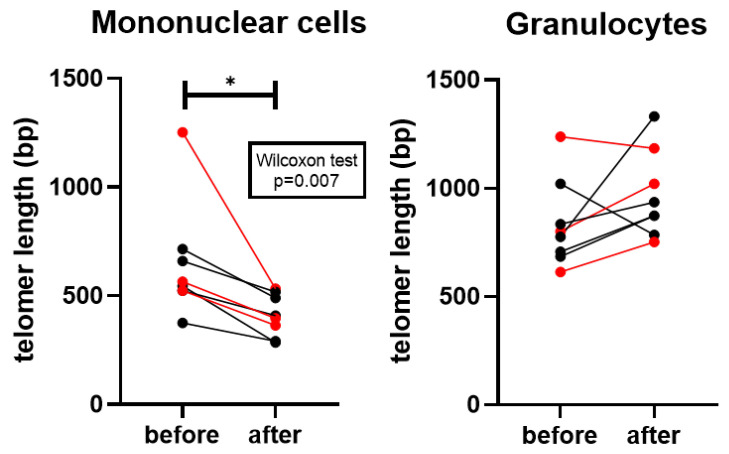
Average telomere length of the diploid cell in mononuclear cells and granulocytes. Red datapoints highlight pembrolizumab-treated patients. * *p* = 0.007.

Average telomere length was significantly shorter in mononuclear cells after vitamin D supplementation (*p* = 0.007), whereas no significant change was observed in granulocytes. Nevertheless, in granulocytes, telomere length showed a non-significant tendency toward elongation, with 6 out of 8 patients exhibiting longer telomeres (except for two lung cancer cases) (Figure 3).

### 3.4. Global DNA Methylation in cfDNA and Blood Cells

**Figure 4:**
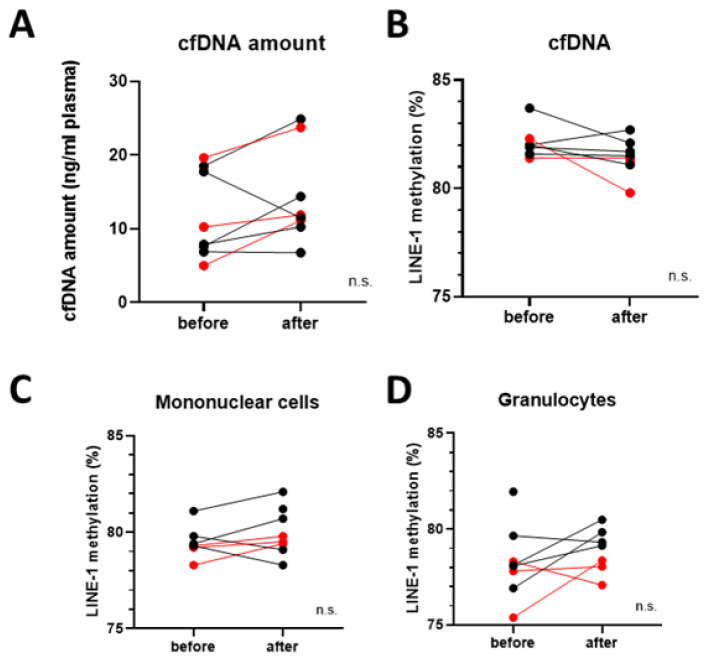
Global DNA methylation analyses. (**A**) cfDNA amount isolated from blood samples before and after vitamin D_3_ supplementation. (**B**) Global DNA methylation levels in cfDNA, (**C**) mononuclear cells, and (**D**) granulocytes before and after vitamin D_3_ supplementation. No significant changes were detected. Red datapoints highlight pembrolizumab-treated patients. n.s. = not significant.

Blood samples were separated into plasma, granulocyte, and monocyte fractions. cfDNA amount isolated from plasma was compared before and after vitamin D_3_ supplementation and slightly, but not significantly elevated levels were detected (Figure 4A).

In order to explore the epigenetic consequences of vitamin D_3_ supplementation, global DNA methylation levels were measured based on LINE-1 retrotransposon methylation levels. No significant difference could be found before and after the supplementation in the cfDNA samples, nor in monocytes and granulocytes (Figure 4B–D). It is visible, that in the granulocytes of the vitamin D + pembrolizumab-treated group had the lowest LINE-1 methylation level after the vitamin D_3_ supplementation period.

### 3.5. Single-Cell ATAC-Seq

**Figure 5:**
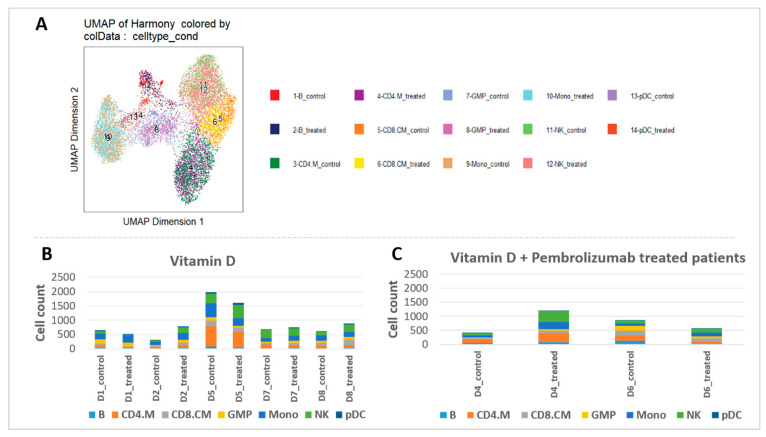
Cell types and cell counts before and after vitamin D_3_ supplementation. (**A**) Cell type identification based on scRNA-Seq data from Granja et al. [36]. (**B**,**C**) Cell counts per cell types before and after vitamin D_3_ supplementation in vitamin D-supplemented and vitamin D + pembrolizumab-treated patients.

To investigate heterogeneous cell populations and to identify unique differences in open chromatin profiles before and after vitamin D_3_ supplementation, single-cell ATAC-Seq was performed. Initially, distinct cell types within the mononuclear cells were identified using reference RNA-Seq datasets. By incorporating pseudo-scRNA-Seq profiles for each scATAC-Seq cell [29] for each scATAC-seq cell, we were able to categorize the immune cell types in our ATAC-Seq results, analyze the ATAC-Seq peaks accordingly, and visualize them using UMAP (Figure 5A, Table S1).

Overall, analysis of the cell ratios among all PBMCs, both before and after supplementation, revealed remarkable differences, particularly in monocytes, NK, and plasmacytoid dendritic cells (pDC) cells (Figure 5B). Patient #3 exhibited minimal cell numbers in the ATAC-Seq results after supplementation and was therefore excluded from the analysis. Basophil granulocytes were detected in the PBMC fraction that can be considered as minor contaminants, likely due to partial overlap in density during gradient separation. After the supplementation period, monocytes increased in 60% of vitamin D supplemented patients (with +26% on average in all patients) and in all vitamin D + pembrolizumab-treated patients (+144%). NK cell numbers increased in 3/5 patients (+69% on average in all patients) in the vitamin D supplemented group, while in 100% in patients who received vitamin D + pembrolizumab (+191%). Additionally, the number of pDC cells increased in 80% of the vitamin D supplemented patients (with +126% on average in all patients) after the vitamin D_3_ supplementation and with ½ of pembrolizumab-treated patients (with +90% on average) (Figure 5C).

**Figure 6:**
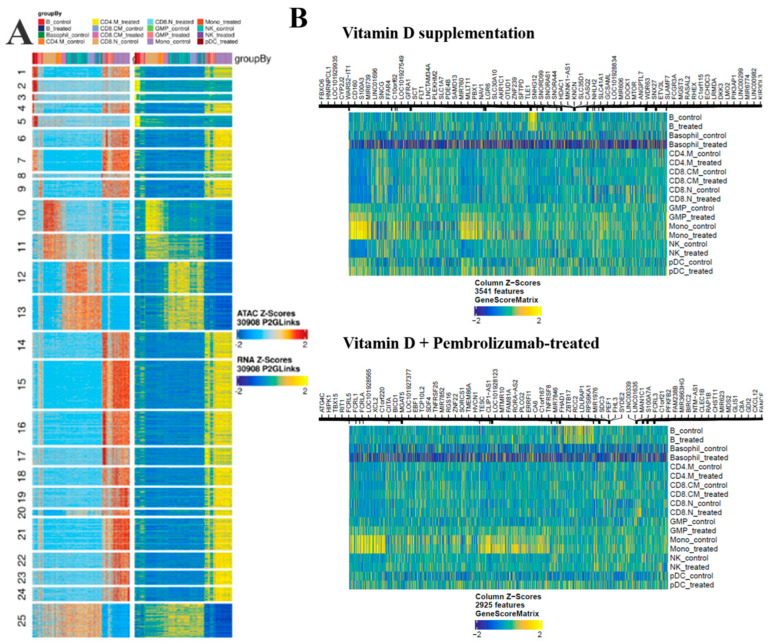
ATAC-Seq heatmaps. (**A**) Schematic alignment of our scATAC-Seq with the reference scRNA-Seq data [29] showing remarkable similarity between the two datasets in the groupwise clusters. The color code of each cell type column can be found at the top of the heatmap panel. (**B**) The identified markers based on Genescore in control and treated cell types in the case of vitamin D supplementation or vitamin D + Pembrolizumab treatment groups.

As a validation attempt, we compared the similarity between our scATAC-Seq data and the applied scRNA-Seq data, and heat map representation was employed (Figure 6A). In 25 clusters, both datasets showed similarity in the control and the supplemented cells. Next, we aimed to identify the genes with differential expressions between the control and the vitamin D-supplemented cells in a cell type-wise manner. Altogether 6079 genes in the vitamin D supplemented group, while 3987 genes were found to be significantly altered in the vitamin D + pembrolizumab-treated patients (FDR ≤ 0.05 & abs(Log2FC) ≥ 1). The majority of the observed alterations were suppression after vitamin D level normalization in the supplemented patients. (Figure 6B, Table S2).

Next, we aimed to perform GO and KEGG analyses to assess the functions affected by the genes with altered chromatin accessibilities. Among the activated functions in the vitamin D supplemented patients, regulation of chromatin organization, telomere organization, Th17 cell differentiation, ncRNA, mRNA processing and metabolic functions, cell proliferation, dendritic cell migration were characteristic, while in the suppressed functions olfactory receptor activity, miRNA-mediated gene silencing were predominant. Among the genes that were altered in vitamin D + pembrolizumab-treated patients, no significant results were found in these analyses (Figure S1).

**Figure 7:**
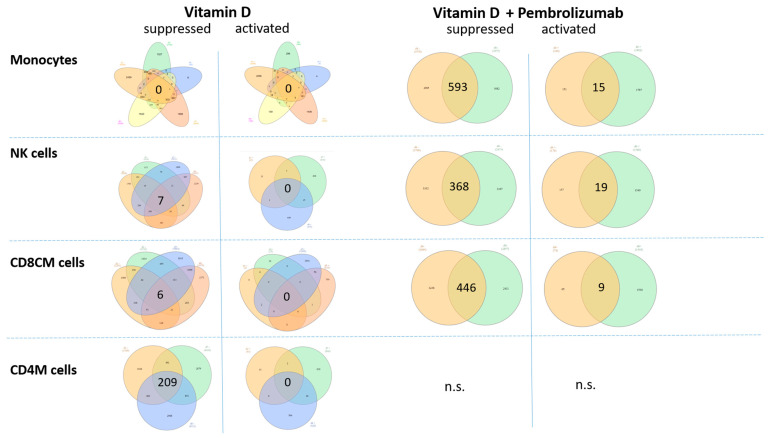
Venn diagram representation of the genes identified with altered chromatin accessibility overlapping between the individuals. Each set represents a patient, with each patient shown in a distinct color. The vitamin D-supplemented and vitamin D + Pembrolizumab-treated patients were separately distributed, focusing on the genes that were suppressed and activated (log2FC ≤ −1 or log2FC ≥ 1). Genes were presented wherever significant results were available from the patients (Table S3). Venn-diagrams were constructed by using the InteractiVenn web-based tool [38]. n.s. = not significant.

To explore the genes that were detected with altered chromatin accessibility, we have tested if there are any common genes in the cell types, grouped on the basis of the applied treatment (if they received pembrolizumab, or not) (log2FC ≤ −1 or log2FC ≥ 1). This analysis revealed certain gene groups that were found in the intersection of the patients’ monocytes, NK cells, CD8CM, and CD4M cells (Figure 7, Table S3).

In general, more genes were found to be overlapping between the patients in the case of the vitamin D + Pembrolizumab-treated group compared to the vitamin D-supplemented groups, which can reflect the clinical manifestation of the immune checkpoint therapy. Interestingly, among the detected chromatin accessibility alterations, suppression was predominant in the vitamin D-supplemented group, while pembrolizumab-treated group contained also activated overlapping genes, as well.

Under vitamin D monotherapy, NK cells showed suppression of pathways associated with tumor-promoting signaling, including genes, such as *FGF19* and *LINC00698*. Additional downregulated genes with limited functional annotation in NK cells (*MAGEA8-AS1*, *KCTD3*, *OR10J5*) likely contribute modestly to this effect. Collectively, these chromatin accessibility changes suggest a slight shift toward reduced tumor-supportive activity in NK cells.

Upon vitamin D supplementation, CD8^+^ central memory (CD8^+^CM) T cells exhibited suppression of pathways related to long-term memory persistence and interferon signaling, reflected by decreased accessibility at genes such as *CD27*, *IFNA1*, and *IRF9*. At the same time, chromatin repression of genes associated with T cell exhaustion and immunosuppressive programs was observed, suggesting a concurrent shift toward a more activated phenotype. Collectively, these patterns indicate a nuanced alteration of CD8^+^CM T cell function, balancing reduced memory-associated signaling with diminished inhibitory pathways.

Vitamin D monotherapy in CD4^+^ memory T cells led to widespread suppression of pathways associated with inhibitory signaling and T cell exhaustion, suggesting an enhanced activation state. However, chromatin accessibility was also reduced in pathways controlling cell proliferation, migration, and survival, indicating that overall functional capacity may be partially attenuated in the absence of checkpoint inhibition.

Following combined vitamin D and pembrolizumab treatment, monocytes exhibited broad chromatin remodeling, with widespread suppression of pathways related to inflammatory signaling, interferon responses, antigen presentation, and Th1-supportive functions, reflected by decreased accessibility at genes such as *CXCL9*, *IL12RB2*, *NKG7*, *TRIM25*, and *GATA3*. Repression of immune-regulatory microRNAs (e.g., *MIR424/503*, *MIR34A*) and epigenetic regulators (*KMT2A*, *HDAC10*) further indicates global attenuation of immune activation programs. In contrast, activation of pathways involved in lipid metabolism and metabolic adaptation (*CYP4F8*, *LDHB*, *PIGV*) suggests that monocytes adopt a metabolically active yet immunologically restrained phenotype, potentially modulating responses to checkpoint therapy while limiting inflammatory toxicity. Collectively, based on chromatin accessibility patterns, these alterations suggest that vitamin D co-treatment may influence monocytes toward a metabolically active but immunologically restrained phenotype, potentially modulating checkpoint therapy responses while limiting inflammatory toxicity.

ATAC-Seq analysis revealed extensive chromatin suppression in NK cells following combined vitamin D and pembrolizumab treatment. Pathways involved in cytokine signaling, effector function, and transcriptional regulation were broadly repressed, as reflected by decreased accessibility at *IL6*, *IL9*, *CCL21*, *CLEC7A*, *TGFB2*, *FOXF2*, *BATF2*, *ARNT*, and regulatory microRNAs (*MIR181C*, *MIR210*), indicating a dampened activation profile. In contrast, pathways related to mitochondrial function and energy metabolism (*XCL1*, *COX7A2*, *ATP5MF*) were activated, suggesting metabolic adaptation. Overall, these chromatin accessibility changes are consistent with a metabolically active yet immunologically restrained NK cell phenotype; however, the absence of a non-supplemented control group precludes direct attribution of these effects to vitamin D.

Following vitamin D supplementation and pembrolizumab treatment, CD8^+^ central memory (CD8^+^CM) T cells exhibited widespread chromatin suppression in pathways related to interferon signaling, antigen receptor co-stimulation, and cytotoxic effector function, as reflected by decreased accessibility at *TYROBP*, *CARD9*, *IRF9*, *CYBB*, *IL17A*, *IFNA1*, *CD27*, and *TNFRSF10A*. Pathways controlling glycolytic metabolism and stress responses (*HIF1A*, *HK2*, *LDHB*) were also downregulated, suggesting attenuated effector metabolism, while reduced accessibility at *AGER* and *LAIR2* indicates dampened immune interaction potential. In contrast, modest activation of pathways associated with metabolic and structural adaptation (*ESX1*, *CADM3*, *PLPP7*, *RETNLB*) was observed, consistent with a shift toward a metabolically quiescent, less inflammatory state rather than classical effector activation. Based on chromatin accessibility patterns, combined supplementation and treatment appear to shift CD8^+^CM T cells toward a less inflammatory, metabolically quiescent state, which may limit immune overactivation under PD-1 blockade while maintaining long-term immune stability. It is important to note that these observations are not derived from direct functional assays and therefore require further investigation.

## 4. Discussion

This work explores a novel direction, which, to the best of our knowledge, has not been attempted before, whereas 8 severe vitamin D insufficient cancer patients were supplemented with an oral vitamin D_3_ dose of 30,000 NE/week and voluntarily gave blood samples before and after 2 months supplementation period. This study primarily aimed to explore the genome-wide vitamin D-dependent chromatin accessibility alterations; therefore, single-cell ATAC-Seq was performed on PBMC cells of the supplemented oncology patients. Furthermore, to assess additional genome-wide epigenetic changes, global DNA methylation analyses were also performed not only on PBMCs and granulocytes, but also on cfDNA samples isolated from plasma samples. Our study included patients treated with Pembrolizumab, an anti-PD-1 inhibitor that restores T cell activity by blocking PD-1/PD-L1 or PD-L2 interactions, reactivating exhausted T cells, and promoting an antitumor immune response [39]. To our knowledge, the present study represents one of the earliest clinical efforts worldwide to investigate the effects of vitamin D level normalization on immune regulation and treatment response in oncology patients undergoing vitamin D_3_ supplementation.

Higher 25(OH)D levels are linked to better prognosis in several cancers and may reduce symptoms such as fatigue, nausea, and vomiting [40,41]. In non-small cell lung cancer, reduced VDR expression can limit vitamin D’s antiproliferative effects [42]. while 1,25-dihydroxyvitamin D regulates apoptosis, proliferation, invasion, and metastasis [43,44,45]. Evidence in lung cancer is inconsistent [46,47], but high VDR expression in the gastrointestinal tract suggests a role for vitamin D in colorectal cancer progression and prognosis [48]. While vitamin D deficiency is frequently observed in cancer patients and supplementation has been associated with improved survival outcomes, the direct immunomodulatory effects of vitamin D in this population remain uncertain, as clinical results are inconsistent [46].

Comet assay is a standard approach for the analysis of oxidation-induced DNA damage leading to mutations and chromosomal instability [49]. Vitamin D_3_ is a potential agent, and its deficiency is associated with a higher frequency of chromosomal aberrations [50], while its supplementation can prevent these alterations by enhancing DNA integrity [51]. Recent experimental evidence indicates that vitamin D reduces DNA fragmentation and micronucleus formation in genotoxic models (e.g., chloramphenicol-induced damage) [52] and protects against DNA damage in human cells in vitro [53]. DNA damage is closely associated with tumor development; for example, cancer patients exhibited significantly higher levels of endogenous DNA damage compared to healthy individuals [54]. Moreover, plasma vitamin D_3_ levels show a significant inverse correlation with the extent of DNA damage in both cancer patients and healthy subjects [54]. Interestingly, patients with vitamin D deficiency showed the highest levels of DNA damage, thereby attributing it as a potential cancer risk factor [54]. According to our results, comet DNA tail percentage in the mononuclear cells of the cancer patients significantly decreased after the supplementation period, which is in line with the abovementioned observations and appears to be a beneficial consequence of vitamin D supplementation.

In our findings, telomere length (TL) was slightly, but not significantly longer in granulocytes after vitamin D level optimization, especially in CRC patients. On the other hand, TL was found to be significantly shorter after vitamin D_3_ supplementation in the mononuclear cells of cancer patients. This is in contrast with several observations examining leukocytes, such as the analysis of Richards et al., where serum vitamin D correlated positively with TL (r = 0.07, *p* = 0.001), even after age adjustment (r = 0.09, *p* < 0.0001) [55]. Unlike most studies that assess telomere length in total leukocytes, our study focused on separated monocytes and granulocytes, which could explain the unexpected finding of shorter telomeres after vitamin D supplementation. On the other hand, since the largest telomere length alteration was observed in the patient who exhibited the highest relative increase in NK cells, we hypothesize that the changes in telomere length may be attributable to shifts in cellular composition following vitamin D_3_ supplementation—for example, the relative increase in NK cells, which are known to harbor shorter telomeres [56,57].

Circulating cfDNA levels are known to be elevated in non-small cell lung cancer (NSCLC) patients compared to those with chronic respiratory inflammation or healthy individuals [58,59], and high cfDNA levels are associated with poorer survival [60,61,62]. Similar patterns have been observed, e.g., in colorectal cancer [63], making cfDNA a promising prognostic biomarker. However, cfDNA levels have rarely been studied in the context of vitamin D_3_ supplementation in cancer patients. In our study, we assessed cfDNA levels during vitamin D_3_ treatment to explore its potential as an indicator of tumor progression or remission. Following the supplementation period, no statistically significant alterations were observed in cancer survival markers, including cfDNA and DNA methylation levels.

Vitamin D has been shown to influence DNA methylation [64]. LINE-1 methylation is a reliable marker of global DNA methylation and is often decreased in various carcinomas including colon, lung, bladder, and prostate cancers [65]. Previous studies have shown that PBMCs from colorectal cancer patients have significantly lower LINE-1 methylation levels compared to controls [66], and cfDNA-based LINE-1 methylation can distinguish lung cancer from inflammatory lung diseases like COPD [67]. In our investigation of vitamin D’s impact on LINE-1 methylation in PBMCs, we could not observe statistically significant alterations; therefore, we assume that while vitamin D may affect DNA methylation, it likely does not cause major changes in global methylation levels.

The number of publications focusing on vitamin D effects on chromatin accessibility is relatively low. For example, in a study of Boutaoui et al., ATAC-seq was used to investigate the genome-wide effects of calcitriol on bronchial epithelial cells [68]. Their results suggest that vitamin D modulates immune cell behavior and gene expression in a way that could influence asthma and viral infections [68]. In another study focusing on the regulatory regions controlling the expression of VDR gene in the intestine by ATAC-Seq [69], researchers found that specific regulatory regions responsible for high expression of the VDR gene in the intestine are conserved between mice and humans [69]. However, the chromatin accessibility of clinical oncology patients after vitamin D_3_ supplementation has been less studied so far.

Vitamin D has been shown to enhance cancer-related immune responses by activating key immune cells such as T cells, dendritic cells, and natural killer (NK) cells, which are essential for recognizing and destroying cancer cells [70,71]. It also modulates inflammation, potentially reducing chronic inflammation that can promote tumor progression [72].

During our chromatin accessibility analyses on PBMC cells, altogether 6079 genes in the vitamin D supplemented group, while 3987 genes were found to be significantly altered in the vitamin D supplemented + pembrolizumab-treated group (FDR ≤ 0.05 & abs(Log2FC) ≥ 1).

On the basis of the publication of Granja et al. [36], scRNA-seq results were combined with our ATAC-Seq data to differentiate each cell type analyzed in our study as the first step of the chromatin accessibility analysis. PBMC cell ratios in cancer patients were altered following vitamin D_3_ supplementation, which may indicate modulation of the immune system. We found that NK cell numbers increased remarkably in both pembrolizumab and non-pembrolizumab-treated groups. Natural Killer (NK) cells play a key role in immune surveillance by recognizing and eliminating tumor cells via cytotoxicity and cytokine secretion [73]. However, in established tumors, NK cell function is often suppressed by immunosuppressive factors like TGF-β, as well as inhibitory ligands (e.g., PD-L1) on tumor cells, leading to immune evasion [74]. Human dendritic cells can be further classified into two subsets of myeloid DCs and plasmacytoid DCs (pDC) [75]. We observed a significant increase in pDC numbers following vitamin D_3_ treatment. It is well established that 1,25(OH)_2_D_3_ plays a distinct immunoregulatory role and exhibits tolerogenic properties, influencing both the maturation and migration of various dendritic cell subsets [76].

We observed elevated monocyte numbers in some treated patients, consistent with reports that 1,25(OH)_2_D_3_ promotes differentiation of normal mononuclear blood cells into the monocyte–macrophage lineage [77].

Analysis of chromatin accessibility across monocytes, NK cells, and T cell subsets revealed that the combined treatment induced a complex response, encompassing both immunostimulatory and potentially immunosuppressive changes. A higher number of overlapping genes was observed between the patients in the vitamin D + pembrolizumab group compared to vitamin D supplementation alone, reflecting the broader immune modulation induced by the checkpoint therapy. Among the common altered genes across patients, suppression predominated in the vitamin D–only group, whereas the pembrolizumab-treated group also showed activated genes, highlighting its immunostimulatory effects. These results indicate that the approach was sensitive enough to detect key alterations consistently present across all patients following supplementation.

Our findings indicate that vitamin D supplementation modulates immune-related gene expression primarily through suppression, while pembrolizumab induces both activation and suppression, reflecting its immune-stimulating effects. The overlap between the two treatments suggests that vitamin D influences some of the same immune pathways as checkpoint therapy. Notably, vitamin D’s suppressive effects may help fine-tune immune responses, potentially reducing overactivation or immune-related adverse events without diminishing pembrolizumab’s therapeutic benefits. This supports the concept that vitamin D could provide complementary immunomodulatory support.

Vitamin D monotherapy produced selective effects across lymphocyte subsets. In NK cells, suppression of tumor-promoting genes indicated reduced tumor-supportive signaling. In CD8 central memory T cells, downregulation of genes such as CD27 and IFNA1 suggested potential compromises in memory persistence, while concurrent suppression of exhaustion-associated genes indicated partial activation. CD4^+^ memory T cells showed widespread suppression of inhibitory genes, pointing to enhanced activation, but downregulation of proliferation and survival genes may limit overall function.

Key favorable effects of vitamin D included enhanced mitochondrial and metabolic gene expression in NK cells, downregulation of suppressive regulators in monocytes and T cells, and increased expression of chemokines that support immune cell recruitment. Potentially detrimental effects involved suppression of effector-supporting genes and possible transcriptional misregulation in specific cell types. Collectively, these data suggest that vitamin D fine-tunes immune responses, mitigating tumor-supportive signaling while modestly promoting activation, complementing—but not replacing—the effects of checkpoint inhibition.

The major limitation of this study is the small number of oncology patients with vitamin D deficiency, specifically eight male patients, who were able to provide blood samples twice within the specified time interval. This non-randomized pre/post study design, coupled with the lack of a separate unsupplemented or vitamin D-sufficient control group, limits the generalizability of the findings and the precise evaluation of the role of vitamin D deficiency in the tumor microenvironment. Additionally, the clinical and therapeutic heterogeneity among participants, including diversity in tumor types and concurrent therapies, makes it challenging to draw definitive conclusions regarding the specific effects of vitamin D supplementation. The influence of these concurrent treatments cannot be fully ruled out, and the observed effects may also be influenced by other unmeasured factors. Another limitation is the short lifespan of PBMCs, which means that baseline and endpoint samples represent different cell populations, a consideration that should be taken into account when evaluating the effects of the two-month treatment.

On the other hand, a major strength of our study is its unique experimental design, in which we specifically recruited participants with clinically confirmed vitamin D deficiency and subsequently restored their serum 25(OH)D levels to the physiological range. This approach allowed us to investigate immune cell responses to vitamin D_3_ supplementation in a controlled, physiologically relevant context, as opposed to studying already sufficient individuals, where additional supplementation might have had a limited biological impact. Furthermore, by applying ATAC-seq, we were able to profile chromatin accessibility changes at high resolution in specific immune cell populations, enabling cell type–resolved analysis of long-term effects of vitamin D level normalization. This integrative approach provides a comprehensive understanding of how vitamin D status directly influences immune regulation at the molecular level and establishes an important foundation for future investigations into its long-term immunomodulatory effects.

The findings of this exploratory study highlight several future perspectives for research. Larger, controlled studies are needed to validate the immunological effects observed here and to account for clinical and therapeutic heterogeneity. Longitudinal investigations with follow-up periods exceeding six months could determine whether the chromatin accessibility and cellular alterations are sustained over time. Incorporating functional assessments, such as NK cell cytotoxicity and cytokine profiling, will be essential to confirm the biological relevance of epigenetic and cellular changes. Finally, clinical trials evaluating oncological outcomes, treatment response, and potential adverse events in patients receiving vitamin D_3_ supplementation could establish its real-world therapeutic impact and inform clinical guidelines. Collectively, these approaches will help clarify the immunoregulatory role of vitamin D in cancer therapy and support the translation of mechanistic findings into clinical practice.

## 5. Conclusions

Vitamin D deficiency is common in cancer patients. This study is the first clinical investigation of the effects of vitamin D_3_ supplementation during conventional chemotherapy and pembrolizumab treatment. Supplementation improved DNA integrity in monocytes, as evidenced by a reduced Comet-tail percentage, and led to significant changes in telomere length in mononuclear cells. Following normalization of vitamin D levels, no clear changes were observed in cfDNA or global DNA methylation. Single-cell ATAC-seq revealed shifts in PBMC composition, particularly in monocytes, NK cells, and plasmacytoid dendritic cells, which may reflect subtle changes in immune function; however, the limited patient cohort precludes strong conclusions. Analysis of chromatin accessibility after combining pembrolizumab and vitamin D treatment showed complex alterations across monocytes, NK cells, and T cell subsets, including both immunostimulatory and potentially suppressive features. These observations may indicate modifications in immune activity, but their interpretation is constrained by the small sample size. These observations are exploratory and hypothesis-generating, and their interpretation is limited by the small patient cohort, the clinical and therapeutic heterogeneity, the absence of a control group, and the lack of direct clinical outcomes. By applying a complex methodological approach, this study provides a framework for future research on the immunoregulatory effects of vitamin D.

## Figures and Tables

**Table 1:** Patient data with tumor diagnosis, stage, oncotherapy, and mutation status, and serum 25(OH)D levels.

ID	Age (Years)	ECOG Status	Tumor	Tumor Stage	Oncotherapy	Mutation Status	Serum 25(OH)D Levels
D1	61	ECOG0	SCLC	pT3N1Mx	partial lobectomy + cisplatin-etoposid + mediastinal irradiation		before: 20.9 ng/mL after: 32.7 ng/mL
D2	68	ECOG0	adenocc pulm	T4N1M0	carboplatin-paclitaxel	TTF1+, KRAS wt, EGFR wt, PDL1 1%, ALK wt, ROS1 wt + Warthin tum.	before: 12.6 ng/mL after: 34.1 ng/mL
D3	60	ECOG1	adenocc. pulm	T3N0M1	pembrolizumab-carboplatin-pemetrexed	KRAS wt, EGFR wt, BRAF wt, ALK neg, ROS1 neg, PDL1 60% + intramuscularis metastases	before: 7.6 ng/mL after: 37.3 ng/mL
D4	50	ECOG1	high grade muscular invasive TCC (transitional cell carcinoma~urothelial cell cc.) + prostata adenocc (prostata apex)	pT3N2M0 + pT1a	neoadjuvant chemotherapy (gemzar + cysplatin-3/3) + opus + Keytruda (pembrolizumab)		before: 20.4 ng/mL after: 36.7 ng/mL
D5	48	ECOG0	CRC rectosigmoidealis régió low grade invasive adenocc	T3N1M0	opus + XELOX		before: 10.7 ng/mL after: 25.7 ng/mL
D6	74	ECOG0	adenocc pulm.	T4N2M1	platina-pemetrexed-pembrolizumab	TTF1 pos, PD-L1 50% pos, KRAS mut, EGFR, ALK neg + intracranialis mpx	before: 6.4 ng/mL after: 32.7 ng/mL
D7	69	ECOG0	carc. adenomatosum invasium colontos	pT4aN1cM0	opus + irradiation + capecitabin		before: 19.5 ng/mL after: 39.8 ng/mL
D8	65	ECOG1	adenocc pulm.	T3N2M0	carboplatin-gemcitabin	PDL1 40%, KRAS p.G12C 22% mut, EGFR neg, BRAF neg, ROS1 neg, ALK neg	before: 8.3 ng/mL after: 58.7 ng/mL

## Data Availability

Due to privacy issues, we do not provide full data availability regarding the sequencing part of this publication. The data are used according to the consent provided by the participants without compromising their anonymity.

## Supplementary Materials

The following supporting information can be downloaded at: https://www.mdpi.com/article/10.3390/nu18010116/s1, Supplementary Figure S1. Altered genes and enriched functions before and after vitamin D_3_ supplementation. Genes with altered chromatin accessibility and GO + KEGG analysis results are illustrated on dotplots for vitamin D-supplemented and vitamin D + pembrolizumab-treated patients; Supplementary Table S1. PBMC cell number alterations after vitamin D supplementation; Supplementary Table S2. Significantly altered genes on the basis of ATAC-Seq in the vitamin D supplemented and the vitamin D supplemented + pembrolizumab-treated groups (FDR ≤ 0.05 & abs(Log2FC) ≥ 1); Supplementary Table S3. Genes with altered chromatin accessibility in the intersection of the similarly treated patient groups.

## Abbreviations

The following abbreviations are used in this manuscript:

PBMC	Peripheral Blood Mononuclear Cells
VDR	vitamin D receptor
NK cells	natural killer cells
pDC cells	plasmacytoid dendritic cells
CYP27B1	Cytochrome P450 family 27 subfamily B member 1
25OHD	25-hydroxyvitamin D
cfDNA	circulating cell-free DNA
ATAC-Seq	assay for transposase-accessible chromatin with sequencing
FAIRE-Seq	Formaldehyde-Assisted Isolation of Regulatory Elements sequencing
ECOG	Eastern Cooperative Oncology Group
SCLC	Small Cell Lung Cancer
CRC	Colorectal Cancer
DPBS	Dulbecco’s Phosphate-Buffered Saline
CpG	Cytosine-phosphate-Guanine
FDR	False Discovery Rate
LINE1	Long Interspersed Nuclear Element-1
UMAP	Uniform Manifold Approximation and Projection
GO	Gene Ontology
KEGG	Kyoto Encyclopedia of Genes and Genomes
CD4M	CD4^+^ memory T cells
CD8CM	CD8^+^ Central Memory T cells
PD/PD-L1	Programmed Death/Programmed Death-Ligand 1
