# Supplementary material for: Modulation of Peripheral Immune Cells Following Vitamin D3 Supplementation in Vitamin D-Insufficient Cancer Patients
Source: Nutrients. 2025 Dec 29;18(1):116. doi: 10.3390/nu18010116 (PMC12788083; doi:10.3390/nu18010116)
Supplement: Supplementary file 1 [file nutrients-18-00116-s001.zip › Figure S1.pdf]

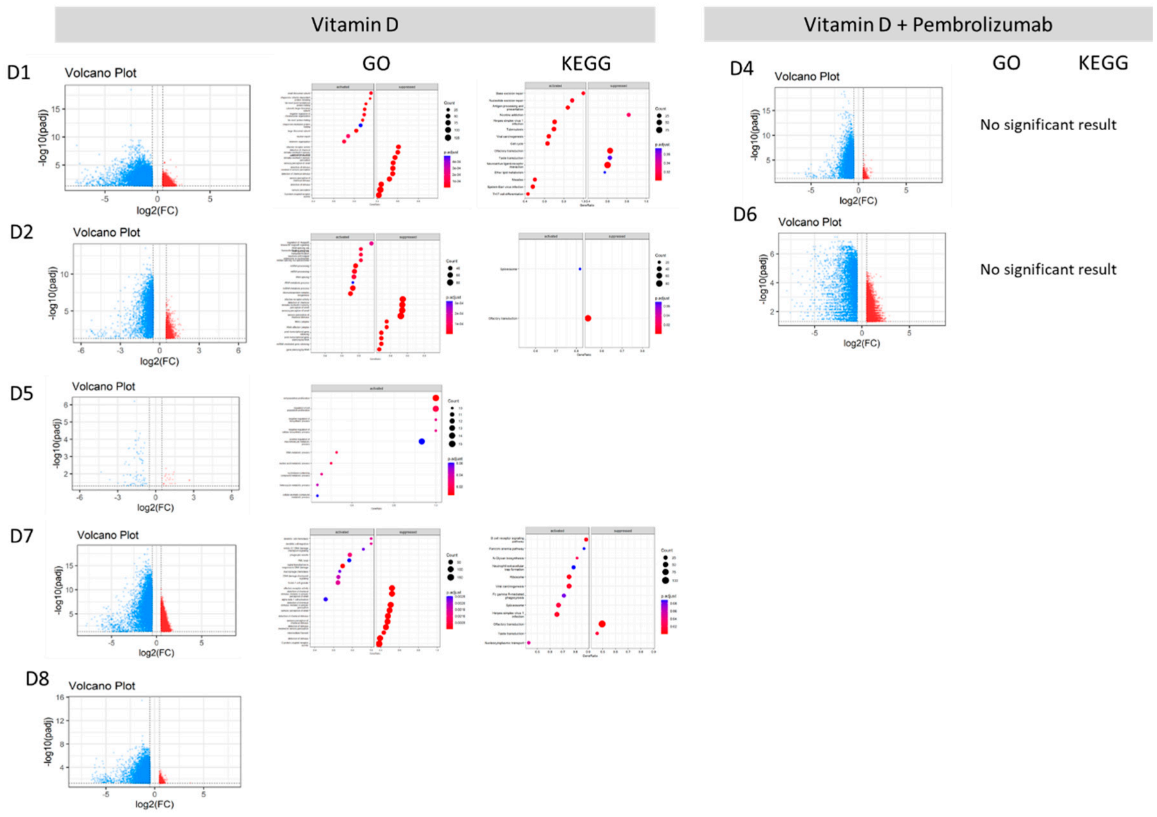


**Supplementary Figure S1. Altered genes and enriched functions before and after vitamin $D_3$ supplementation.** Differentially expressed genes and GO + KEGG analysis results are illustrated on dotplots for vitamin D-supplemented and vitamin D + pembrolizumab-treated patients.
